# Global Role of Cyclic AMP Signaling in pH-Dependent Responses in *Candida albicans*

**DOI:** 10.1128/mSphere.00283-16

**Published:** 2016-11-30

**Authors:** Jeffrey M. Hollomon, Nora Grahl, Sven D. Willger, Katja Koeppen, Deborah A. Hogan

**Affiliations:** Department of Microbiology and Immunology, Geisel School of Medicine at Dartmouth, Hanover, New Hampshire, USA; Carnegie Mellon University

**Keywords:** *Candida albicans*, Cyr1, Ras1, Rim101, adenylate cyclase, morphology, pH

## Abstract

*Candida albicans* behaviors are affected by pH, an important environmental variable. Filamentous growth is a pH-responsive behavior, where alkaline conditions favor hyphal growth and acid conditions favor growth as yeast. We employed filamentous growth as a tool to study the impact of pH on the hyphal growth regulator Cyr1, and we report that downregulation of cyclic AMP (cAMP) signaling by acidic pH contributes to the inhibition of hyphal growth in minimal medium with GlcNAc. Ras1 and Cyr1 are generally required for efficient hyphal growth, and the effects of low pH on Ras1 proteolysis and GTP binding are consistent with diminished cAMP output. Active alleles of *ras1* do not suppress the hyphal growth defect at low pH, while dibutyryl cAMP partially rescues filamentous growth at low pH in a *cyr1* mutant. These observations are consistent with Ras1-independent downregulation of Cyr1 by low pH. We also report that extracellular pH leads to rapid and prolonged decreases in intracellular pH, and these changes may contribute to reduced cAMP signaling by reducing intracellular bicarbonate pools. Transcriptomics analyses found that the loss of Cyr1 at either acidic or neutral pH leads to increases in transcripts involved in carbohydrate catabolism and protein translation and glycosylation and decreases in transcripts involved in oxidative metabolism, fluconazole transport, metal transport, and biofilm formation. Other pathways were modulated in pH-dependent ways. Our findings indicate that cAMP has a global role in pH-dependent responses, and this effect is mediated, at least in part, through Cyr1 in a Ras1-independent fashion.

**IMPORTANCE** *Candida albicans* is a human commensal and the causative agent of candidiasis, a potentially invasive and life-threatening infection. *C. albicans* experiences wide changes in pH during both benign commensalism (a common condition) and pathogenesis, and its morphology changes in response to this stimulus. Neutral pH is considered an activator of hyphal growth through Rim101, but the effect of low pH on other morphology-related pathways has not been extensively studied. We sought to determine the role of cyclic AMP signaling, a central regulator of morphology, in the sensing of pH. In addition, we asked broadly what cellular processes were altered by pH in both the presence and absence of this important signal integration system. We concluded that cAMP signaling is impacted by pH and that cAMP broadly impacts *C. albicans* physiology in both pH-dependent and -independent ways.

## Abstract (precis)

*Candida albicans* is a human commensal and the causative agent of candidiasis, a potentially invasive and life-threatening infection. *C. albicans* experiences wide changes in pH during both benign commensalism (a common condition) and pathogenesis, and its morphology changes in response to this stimulus. Neutral pH is considered an activator of hyphal growth through Rim101, but the effect of low pH on other morphology-related pathways has not been extensively studied. We sought to determine the role of cyclic AMP signaling, a central regulator of morphology, in the sensing of pH. In addition, we asked broadly what cellular processes were altered by pH in both the presence and absence of this important signal integration system. We concluded that cAMP signaling is impacted by pH and that cAMP broadly impacts *C. albicans* physiology in both pH-dependent and -independent ways.

## INTRODUCTION

Local pH is one facet of the environment that varies widely in the human host-associated niches occupied by *Candida albicans*, a commensal fungus and opportunistic pathogen. Benign chronic carriage of *C. albicans* in the gastrointestinal tract occurs frequently, and over the length of this organ system, the pH ranges from 2 in the stomach to 8 in the large intestine and can vary widely from subject to subject (1). Additionally, the vagina is acidic during benign *C. albicans* carriage and *Candida* vaginitis (2). We and others have shown that endogenous fermentative metabolism can substantially reduce the extracellular pH in sugar-rich environments, particularly in situations where respiratory metabolism is limited (3, 4). Filamentous growth, a pathogenesis-related trait, is pH sensitive *in vitro*, with the number of hyphae in the population increasing over the pH range from 5 to 7 (5).

*C. albicans* pH sensing and its morphological response to pH are in part controlled by the PacC ortholog Rim101, a transcription factor that is posttranslationally activated in response to elevated pH (6). Rim101 is a major contributor to *in vitro* hyphal growth in rich, high-pH tissue culture medium (M199 at pH 8) (7), and disruption of the pH-sensing regulator Rim101 results in striking virulence defects in murine models of disseminated and oropharyngeal candidiasis and *Candida* keratomycosis (8–10). However, Rim101 is not absolutely required for hyphal growth; it is dispensable for the formation of hyphae in response to serum, which is thought to activate hyphal growth through the cyclic AMP (cAMP) pathway.

Ras1 and adenylate cyclase (Cyr1) are central components of cAMP signaling. They work together to govern filamentous growth, responses to stress, coordination of multicellular behaviors, and white-opaque switching in *C. albicans* (reviewed in reference 11), and both Ras1 and Cyr1 contribute to virulence in a murine model of disseminated candidiasis (12, 13). Ras1 is a soluble small GTPase which, in its GTP-bound conformation, activates the adenylate cyclase Cyr1 (also referred to as Cdc35 in the literature) (14). The resultant increase in intracellular cAMP relieves repression of protein kinase A by its regulatory subunit Bcy1, resulting in the initiation of the hyphal growth program (15–17). This pathway integrates a multitude of environmental signals, and induction of hyphal growth in response to a number of stimuli has been shown to involve the Ras1/cAMP pathway, notably serum, GlcNAc, muramyl dipeptides, and elevated temperature (18–21). Findings from our group, however, have called into question the canonical unidirectional and linear relationship where stimuli act on Ras1 to activate Cyr1. We have shown Cyr1 to act upon Ras1 by negatively regulating both the as-yet-unidentified protease that alters Ras1 localization and the Ras1 GTPase-activating protein Ira2 (22, 23). Ras1-independent activation of Cyr1 leads, through repression of Ras1 cleavage, to reinforcement of Cyr1 activation. Conversely, Cyr1 inhibition of Ira2 negatively regulates the activation of Cyr1 by Ras1 in response to cascade output. Synthesis of these observations yields a model where the relationship between Ras1 and Cyr1 is complex and nonlinear, in which Ras1 can activate Cyr1 in response to some stimuli but Ras1 activation is in turn tightly regulated by Cyr1.

Here, using a citrate-buffered defined medium that can be poised at either pH 4 or 7, we confirmed that more cells had hyphal morphology at neutral pH, whereas pseudohyphae and yeast predominated at pH 4. As these hypha-inducing conditions (37°C with GlcNAc) activate Ras1 and Cyr1 (21, 24) and true hyphal growth is suppressed in this medium by low pH, we asked if Ras1 or Cyr1 is downregulated in response to low pH, and if so, whether the reduction in activity of this pathway contributes to the observed repression of hyphal growth at low pH. We found that the fraction of Ras1 in the proteolyzed form and the fraction of Ras1 in its active GTP-bound conformation increase at low pH. These changes in Ras1 require Cyr1, which we have shown previously to negatively regulate both Ras1 cleavage and Ras1-GTP levels. Thus, we propose that low pH downregulates Cyr1 activity, and this is consistent with the observation that dibutyryl cyclic AMP restores hyphal growth in a *cyr1*Δ/Δ mutant strain at both pH 4 and pH 7 (22, 23). A transcriptional analysis revealed pH-dependent and -independent effects of Cyr1 on gene expression.

## RESULTS

### Low pH represses growth morphology of hyphae and promotes growth as pseudohyphae in inducing medium with GlcNAc.

**FIG 1:**
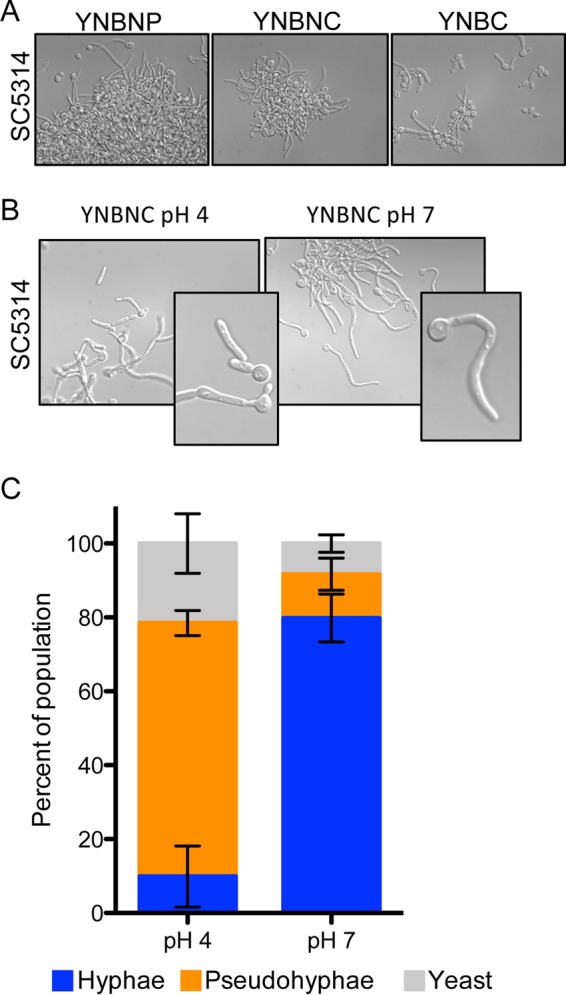
Low pH represses growth of cells with the hyphal morphology and promotes growth as pseudohyphae. (A) GlcNAc promotes hyphal induction in citrate-buffered medium. Representative images show cells grown in the indicated medium for 3 h at 37°C in YNB or YNBN medium buffered to pH 7 with phosphate or citrate, and counts are from 3 independent experiments. (B and C) Medium buffered to pH 4 promoted pseudohyphal growth. Representative images of cells grown for 3 h in YNBNC at pH 4 and 7 are shown (B), and quantitative enumeration of cellular morphologies is summarized (C). Error bars represent one standard deviation.

In order to study the effects of pH on *C. albicans*, we sought to implement a buffer system for use in a defined, hypha-inducing medium that would support analysis of effects of pH on morphology. To achieve this, we used a variation of YNBNP (yeast nitrogen base, *N*-acetylglucosamine, phosphate; a defined medium with GlcNAc and buffered to pH 7 with phosphate) in which the phosphate buffer was replaced with citrate buffer (YNBNC). Citrate has pK_a_’s at 3.1, 4.8, and 6.4, which make it possible to study the effects of low and neutral pH with a single buffer. Following inoculation of *C. albicans* SC5314 wild-type (WT) cells from an overnight culture grown as yeast in yeast extract-peptone-dextrose (YPD) at 30°C into YNBNC (pH 7) at 37°C, a majority of cells formed true hyphae (92% ± 4% [mean ± standard deviation]), with no invagination at either the bud neck or septa along the germ tube at the 3-h time point, similar to results obtained in the phosphate-buffered variant of this medium (YNBNP), though the percentage of cells growing as true hyphae was slightly less than in medium with a phosphate buffer at this time point (Fig. 1A). In YNBC without GlcNAc at 37°C, fewer than 25% of cells grew as true hyphae, suggesting that GlcNAc plays a role in the induction of filamentation in this medium.

In a separate set of experiments, we compared cells grown in YNBNC buffered to either pH 7 or 4 at 37°C for 3 h, and hypha formation by wild-type SC5314 was 80% ± 6% of total cells at pH 7 and 10% ± 8% of cells at pH 4. At the lower pH, the dominant population was pseudohyphae (68% ± 3% of total cells) (Fig. 1B and C). This finding is consistent with previously published observations in which filamentous growth is antagonized by medium pH values below ~6 (5, 7, 25). To determine if the lack of filamentation under the pH 4 culture conditions was due to inhibition of growth, we compared growth rates at the two different pHs and assessed growth by using the yeast-locked *tetO-NRG1* strain (bearing a tetracycline-repressible copy of the *NRG1*-encoded hyphal repressor [26]) that enabled growth analysis by measurement of light absorbance. We found that growth was not lower but in fact slightly higher at pH 4 than at pH 7. In YNBNC at 37°C, this strain had a doubling time of 102 ± 1 min at pH 4 and 112 ± 3 min at pH 7. To rule out the possibility that *NRG1* overexpression was affecting growth, we also examined growth of WT SC5314 at pH 4 and pH 7 at 30°C in YNBC, which is identical to YNBNC except it lacks the hyphal growth inducer GlcNAc. Again, growth was slightly but significantly faster at pH 4 than at pH 7 (113 ± 1 min and 120 ± 6 min, respectively). In both cases, the slightly faster growth at pH 4 was statistically significant (*P* < 0.05).

### Disruption of Cyr1 and Ras1 results in an inability to grow as hyphae at pH 4 and 7.

**FIG 2:**
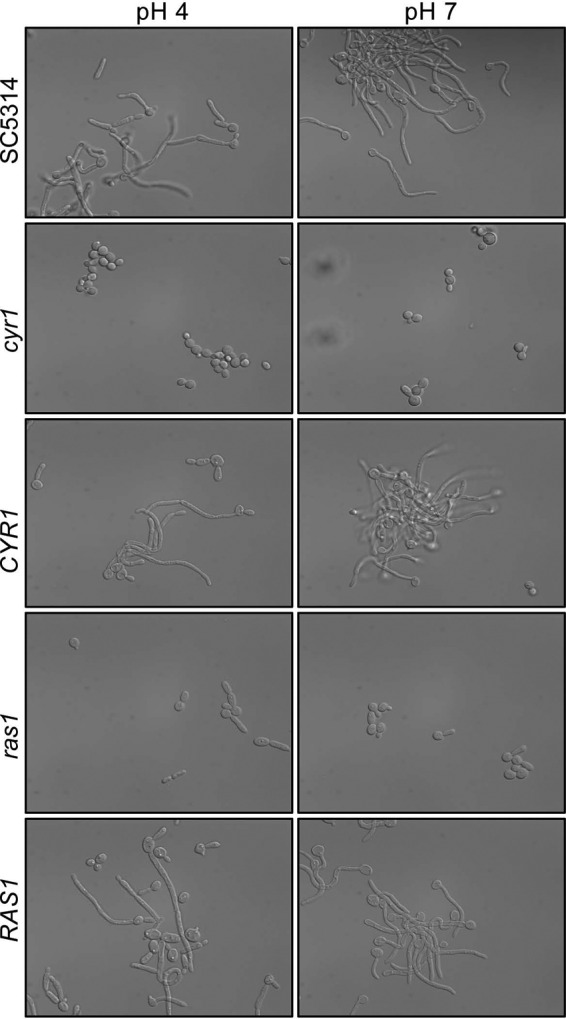
Disruption of Cyr1 and Ras1 results in an inability to grow as hyphae at pH 4 and 7. Ras/cAMP signaling is required for efficient induction of filamentation in YNBNC. Strains were photographed following 3 h of growth at 37°C in YNBNC at pH 4 or 7.

Cyr1 and Ras1 work together to positively regulate filamentous growth in response to GlcNAc and elevated temperatures in liquid medium, and mutants lacking the genes encoding either of these proteins are impaired in hyphal growth (12, 19, 21). The adenylate cyclase Cyr1 has been described as essential for filamentation in liquid medium, and when morphology was assessed over the course of 24 h, *cyr1Δ/Δ* mutants grew exclusively as yeast in YNBNC at either pH 7 or pH 4, whereas the WT and the *cyr1Δ/Δ*::*CYR1* mutant strain formed predominantly pseudohyphae at pH 4 and hyphae at pH 7 (Fig. 2) (13). Consistent with previous studies that have shown that strains lacking *RAS1* have a less severe defect than do *cyr1* null mutants, a *ras1*Δ/Δ mutant strain grew as a combination of pseudohyphae and yeast (Fig. 2). Reconstitution of a wild-type allele of *RAS1* at the native locus restored growth similar to that of the wild type as true hyphae at pH 7, but cells still had pseudohyphal morphology at pH 4 (Fig. 2).

### Low pH induces Ras1 proteolysis, but this does not explain the change in morphology.

**FIG 3:**
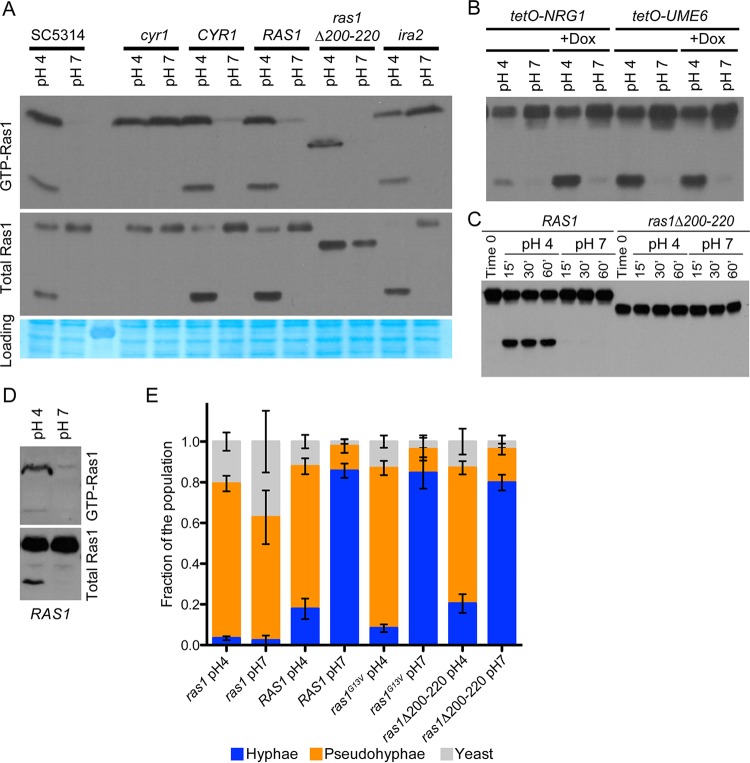
Low pH alters Ras1 localization and its activation state, but these changes are not responsible for the effect of pH on morphology. (A) Total Ras levels (top) were comparable at pH 4 and 7, although cleavage prevailed at pH 4. (Top) Western blot results for the GTP-bound fraction of the lysates. (Middle) Anti-Ras1 Western blot results with whole-cell lysates. (Bottom) Gelcode Blue stain of the membrane blotted in the top panel, to indicate loading. Cells were grown for 3 h in YNBNC at the indicated pH. (B) Western blot of the Ras1 profile and microscopy of *tetO-UME6* and *tetO-NRG1* strains with and without 50 µg per ml doxycycline. (C) Ras1 profile of cells of the indicated genotype grown for 3 h in YNB–GlcNAc–0.2% glucose (YNBNG) adjusted to pH 7 and subsequently buffered to the indicated pH with citrate for the indicated time. (D) Ras1-GTP binding of *RAS1* cells grown for 3 h in YNBNG adjusted to pH 7 and subsequently buffered to the indicated pH with citrate for 30 min. (E) Quantitative morphology of the indicated strains grown for 3 h in YNBNC at 37°C at pH 4 or pH 7. Error bars represent one standard deviation.

Cyr1 responds to a variety of Ras1-dependent and -independent signals (reviewed in reference 27). Ras1 and Cyr1 reciprocally regulate each other; Cyr1 activity can be stimulated by Ras1-GTP, and Ras1-GTP binding and Ras1 proteolysis are negatively regulated by Cyr1 (22, 23). To determine if Ras1 total protein levels differed in cells grown in YNBNC at either pH 7 or pH 4 (22), Ras1 abundance was measured by Western blotting. In SC5314, and also the *ras1Δ/Δ*::*RAS1* strain expressing native Ras1, total Ras1 protein levels (the sum of cleaved and full-length Ras1) were not different between cells grown at pH 4 versus pH 7. However, growth at pH 4 resulted in a decrease in full-length Ras1 concomitant with the appearance of the cleaved product, with 48% ± 12% of the total Ras1 existing in the cleaved isoform at pH 4 and no consistently detectable cleaved Ras1 at pH 7 (Fig. 3A). A strain bearing only a *RAS1* allele in which the 20 residues containing the cleavage site have been deleted (*ras1Δ200-220*) produces a Ras1 protein that is not cleaved, and it supports robust hyphal growth (22). In the *ras1Δ200-220* strain, Ras1 was uncleaved at pH 4 and pH 7 and did not show the same decrease in full-length Ras1 as the cleaved product that accumulated in SC5314 or an isogenic strain bearing the WT allele (Fig. 3A).

To determine if the effect on Ras1 proteolysis was downstream of two important transcriptional regulators of morphology, we interrogated the Ras1 profile of the *tetO-NRG1* and *tetO-UME6* strains, which overexpress a hyphal repressor (*NRG1*) or a hyphal activator (*UME6*) in the absence of doxycycline (Fig. 3B). In the *tetO-UME6* strain grown without doxycycline, the cleaved product was more abundant at pH 4 than pH 7, suggesting that alterations in the Ras1 protein profile are not downstream or independent of these transcriptional regulators. In the presence of *NRG1* overexpression, the induction of proteolysis also took place in response to pH, though proteolysis was induced less strongly, suggesting Nrg1 as an indirect or direct regulator of Ras1 proteolysis.

### Ras1-GTP binding increases following exposure to low pH.

As the effect of low pH on morphology is consistent with downregulation of Ras signaling, we sought to determine the effect of pH on Ras1-GTP binding. The fraction of Ras1 in its active GTP-bound state was assayed by precipitation with recombinant Ras binding domain (RBD) followed by elution and detection by Western blotting with an anti-Ras antibody. The proportion of the total Ras1 pool that was GTP bound was markedly higher in cells grown at pH 4 than in those at pH 7, with both the full-length and cleaved isoforms showing increased GTP binding (Fig. 3A). Ras1 cleavage is not responsible for the increase in GTP binding, as increased Ras1-GTP binding at low pH was also observed in a strain with the *ras1Δ/Δ*::*ras1Δ200-220* genotype.

### The effects of low pH on Ras1 cleavage and GTP binding are rapid.

The effects of the pH shift on Ras1 were rapid as well as sustained. When cells were grown at neutral pH, we observed the appearance of the Ras1 cleavage product within 15 min after cells were shifted to pH 4 with citrate buffer, but not when cells were transferred to a medium buffered to pH 7 with citrate. Furthermore, this difference in Ras1 profiles between pHs persisted over the course of growth and was stable for an hour following the shift to pH 4 from pH 7, suggesting that the alteration of Ras1 cleavage by pH is fast as well as persistent (Fig. 3C). The increase in Ras1-GTP binding was also rapid and was evident within 30 min of a shift to low pH (Fig. 3D).

### Ras1 proteolysis and GTP binding are not responsible for pseudohyphal morphology at pH 4.

Above, we reported that low pH increases Ras1 cleavage and Ras1-GTP binding. Ras1 proteolysis delocalizes the Ras1 N-terminal catalytic domain from the plasma membrane and decreases Ras1- and Cyr1-dependent hyphal growth (22). To determine if pH-induced cleavage of Ras1, and the resultant decrease in the amount of Ras1 at the membrane, is responsible for the decreased growth as hyphae at pH 4, we assessed morphology of the WT *RAS1* strain and the *ras1Δ200-220* mutant strain in cultures grown at pH 4 and 7. The ability to cleave Ras1 did not affect the proportion of the population growing as pseudohyphae at low pH; the *ras1Δ/Δ*::*RAS1* and *ras1Δ/Δ*::*ras1Δ200-220* mutant strains similarly grew as hyphae at pH 7 and pseudohyphae at pH 4, with no statistically significant difference in the ratio of hyphae to pseudohyphae between the two strains at pH 4 or pH 7 (Fig. 3E).

Although hyperactivation of cAMP signaling frequently results in hyphal growth, it has also been reported to result in pseudohyphae in place of hyphae under filamentation-inducing conditions (12, 28). To determine if the increase in GTP binding was responsible for pseudohyphal growth at pH 4, we compared the morphology of the *ras1*Δ/Δ::*ras1G13V* mutant (bearing an allele that encodes a constitutively GTP-bound Ras1) to that of the *ras1*Δ/Δ::*RAS1* mutant. The *ras1Δ/Δ*::*ras1G13V* mutant and its isogenic *ras1Δ/Δ*::*RAS1* comparator mutant strain behaved like the wild type and were similarly pseudohyphal at pH 4 and hyphal at pH 7 (Fig. 3E). These data indicate that changes in Ras1 cleavage and GTP binding are not responsible for the decrease in hyphal growth at pH 4.

### Effects of pH on Ras1 require Cyr1, and dibutyrl cAMP (dbcAMP) can rescue hyphal growth at low pH.

Work from our group has previously shown that Cyr1-synthesized cAMP represses Ras1 proteolysis in hyphae (22). Additionally, we have found that diminution of Cyr1 activity results in increased Ras1-GTP binding through inactivation of Ira2 (23). In an *ira2-*deficient strain, which lacked the gene encoding the Ras1 GTPase-activating protein, Ras1 was proteolyzed in response to low pH but had high and pH-insensitive Ras1-GTP binding, indicating that the increase in GTP binding in response to pH is mediated through Ira2 (Fig. 3A). Based on this observation, we concluded that the increase in proteolysis at low pH was not solely due to the increase in Ras1-GTP levels. Moreover, as with Ras1 proteolysis, the increase in GTP binding occurred following exposure of preformed hyphae grown at neutral pH to low pH (Fig. 3D).

**FIG 4:**
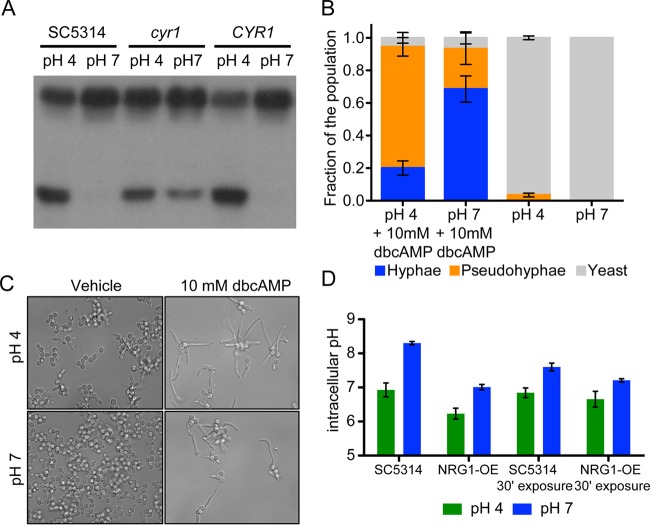
Alterations in morphology, Ras1 cleavage, and intracellular pH are consistent with pH inhibition of cAMP signaling. (A) Anti-Ras1 Western blot results with whole-cell lysate of cells grown for 3 h in YNBNC at the indicated pH. (B) Quantitative morphology of *cyr1*Δ/Δ strain cells grown for 3 h at 37°C in YNBNC with or without 10 mM dbcAMP. (C) Representative images of cells grown as described for panel B. (D) pHluorin-determined intracellular pH of cells of the indicated genotype grown for 3 h in YNBNC at 37°C at pH 4 or 7 (left) or grown in YNBN for 3 h and subsequently shifted to pH 4 or 7 with 50 mM citrate buffer for 30 min (30′ exposure; right). Error bars in panels B and D represent one standard deviation.

Based on the known Cyr1 repression of levels of proteolyzed Ras1 and Ras1-GTP, and the observed increase in both of these Ras1 forms in cells at low pH, we hypothesized that low pH inhibits Cyr1 in a Ras1-independent fashion. To test this hypothesis, we examined the effect of pH on Ras1 proteolysis and GTP binding in the presence and absence of Cyr1. Consistent with this model, we found that the induction of Ras1 proteolysis and GTP binding in response to low pH was substantially diminished in the *cyr1* null strain (Fig. 3A and 4A). Our data indicated that the absence of the cleaved product from the input blot in the GTP binding assay in the *cyr1* mutant was due to postlysis degradation in the Ras1-GTP pulldown buffers; the cleaved product was present when lysates were prepared in homogenization buffer (defined in Materials and Methods) (see Fig. S1 in the supplemental material). The basis of the difference in the stability of the Ras1 cleaved product between the *cyr1* null strain and its complement is not yet understood. To test the hypothesis that this event took place as a result of a change in the output of the Rim101 pathway, we examined the Ras1 cleavage profile of cells shifted to pH 4 or maintained at pH 7, with and without *RIM101*. The induction of cleavage was observed in *rim101Δ/Δ* strains, indicating that regulation of this proteolytic event by pH is independent or upstream of the Rim101 cascade (see Fig. S2).

10.1128/mSphere.00283-16.1 Figure S1  Effect of lysis buffer on the stability of cleaved Ras1 product. Cells were grown in YNBNC at the indicated pH for 3 h and lysed via beadbeating in either homogenization buffer or lysis-binding-wash buffer from the Active Ras1 pulldown kit (Fisher Scientific). Download Figure S1, JPG file, 0.3 MB. Copyright © 2016 Hollomon et al. 2016 Hollomon et al. This content is distributed under the terms of the Creative Commons Attribution 4.0 International license.

10.1128/mSphere.00283-16.2 Figure S2  Western blot analysis of Ras1 profiles of cells of the indicated genotype grown for 3 h in YNBN, adjusted to pH 7 at 37°C, and shifted to the indicated pH with 50 mM citrate buffer. Download Figure S2, JPG file, 0.2 MB. Copyright © 2016 Hollomon et al. 2016 Hollomon et al. This content is distributed under the terms of the Creative Commons Attribution 4.0 International license.

We and others have observed that the nonhydrolyzable, cell-permeable cAMP analog dbcAMP is able to rescue the phenotypic effects of reduced cyclic AMP signaling, and we hypothesized that addition of dbcAMP would suppress the effect of pH on morphology under our conditions (12, 13, 22, 24). Addition of dbcAMP was able to induce filamentous growth at both pH 4 and pH 7 in a *cyr1* null strain (Fig. 4B and C). At pH 4 with 10 mM dbcAMP, 20% ± 4% of the population grew as true hyphae, 74% ± 6% of the population grew as pseudohyphae, and the remainder grew as yeast, whereas in the absence of dbcAMP all cells were in the yeast form. This indicated to us that addition of dbcAMP was able to partially overcome the inhibitory effect of low pH on filamentous growth. The addition of dbcAMP also stimulated hyphal growth at pH 7, whereas in the presence of dbcAMP, 68% ± 8% of the population grew as true hyphae, 25% ± 10% of the population grew as pseudohyphae, and the rest grew as yeast; at pH 7 without dbcAMP, cells similarly grew as yeast. The observation that at pH 4 a *cyr1* null strain is capable of both hyphal and pseudohyphal growth in the presence of dbcAMP suggests that the effect of pH on filamentous growth, at least in part, requires Cyr1. Taken together, these data are consistent with the model that low pH inhibits cAMP synthesis by Cyr1 independently of Ras1 to negatively regulate hyphal growth in response to acidic pH.

### pH of the medium affects intracellular pH in YNBNC.

Work in the Mühlschlegel lab identified bicarbonate as an important activator of Cyr1 in *Candida albicans* (29). At physiological pH, bicarbonate acts as a buffer and exists in equilibrium with its conjugate acid, carbonic acid. As our data indicated that Cyr1 output decreases at low pH, we hypothesized that growth at low pH resulted in a lower cytosolic pH, which could drive the equilibrium from bicarbonate toward carbonic acid, thereby depleting this activator of Cyr1.

To test the hypothesis that the pH of the medium alters the cytosolic pH under our conditions, we employed a *Candida* optimized, pH-sensitive ratiometric green fluorescent protein (GFP; pHluorin) (30), which is predictably altered in its fluorescence emission wavelength by pH. SC5314 cells expressing pHluorin grown in YNBNC at pH 7 for 3 h had an intracellular pH (pH_i_) of >8, over 1.1 units higher than cells grown in the same medium at pH 4 (pH_i_ 6.9) (Fig. 4D). Cells grown as hyphae at neutral pH and subsequently shifted to pH 4 with citrate also showed a reduction in pH_i_ compared to controls maintained at pH 7 with citrate. We measured the pH_i_ for the *tetO-NRG1* strain, where although the cytosol was more acidic under each condition compared to the wild type, the pH_i_ was still lower in cells grown at pH 4 than in those grown at pH 7.

### Definition of the Cyr1 regulon under acidic and neutral medium conditions.

**FIG 5:**
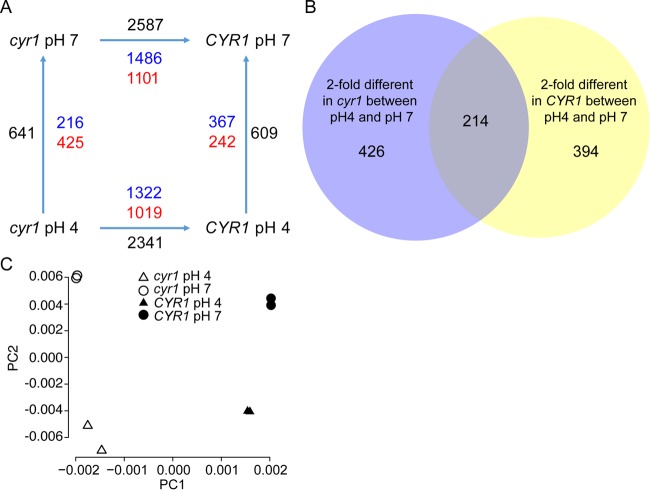
RNA-Seq analysis of *CYR1* cells in the adaptation to acid pH. (A) Black shows the sum of transcripts that were >2-fold different and had an FDR of <0.05; blue shows transcripts that were upregulated at pH 7 and complemented; red shows transcripts that were downregulated at pH 7 and complemented. (B) Overlap of genes differentially regulated by pH in the presence and absence of strain *CYR1*. (C) Principal component analysis of the *cyr1Δ/Δ* mutant and *CYR1* strain samples at pH 4 and pH 7.

With evidence that low pH affects cAMP signaling and pH_i_, we employed an unbiased transcriptomic approach to determine the contribution of *C. albicans* Cyr1 to differences in gene expression between neutral and low pH. We grew both the *cyr1Δ/Δ* mutant and its complemented derivative for 4 h at either pH 4 or 7, followed by RNA extraction from cells and transcriptome sequencing (RNA-Seq) analysis. At pH 7, the *cyr1Δ/Δ* mutant had many genes that were differentially expressed relative to its complemented strain, with 2,587 transcripts (1,101 upregulated and 1,486 downregulated in the mutant compared to the complemented strain) meeting our criteria for significantly different expression (>2-fold change, <0.05 false-discovery rate [FDR]) (Fig. 5A; see also Table S2 in the supplemental material). There were similarly large differences between the *cyr1Δ/Δ* strain and its complement at pH 4, with 2,341 significantly different transcripts (1,019 increased and 1,322 decreased) (Fig. 5A). Across both pH 4 and pH 7, 1,808 transcripts were significantly changed in the absence of Cyr1 (771 increased and 1,037 decreased at pH 7, 769 increased and 1,039 decreased at pH 4, and 16 transcripts lower at one pH and higher at the other). Comparison of the differentially expressed genes between the *cyr1* mutant and the *CYR1* complemented strain to the Cyr1 regulon defined by Harcus et al. in 2004 (31) found 196 transcripts (88 positively regulated and 108 negatively regulated by Cyr1) that were differentially expressed in both data sets (Table S3), and they included transcripts associated with hyphal growth, including *ECE1*, *HWP1*, *HYR1*, *SAP4*, and *SAP6*. In our data, the yeast-locked *cyr1* mutant also had lower levels of other hypha-associated transcripts *UME6*, *RBT1*, *ALS3*, *SAP5*, *IHD1*, and *HGC1* and higher levels of the yeast-related transcripts *YWP1*, *ALS4*, and *NRG1* (Table 1). Analysis by gene ontology (GO) term enrichment of the Cyr1 regulon at pH 7 and pH 4 revealed an enrichment in genes annotated as having a role in biofilm formation, which includes many of the morphology-associated genes (*HWP1*, *HYR1*, *ECE1*, and *HGC1*) (Table 2; Table S5).

10.1128/mSphere.00283-16.3 Table S1  Strains used in this study. Download Table S1, DOCX file, 0.1 MB. Copyright © 2016 Hollomon et al. 2016 Hollomon et al. This content is distributed under the terms of the Creative Commons Attribution 4.0 International license.

10.1128/mSphere.00283-16.4 Table S2  Transcripts meeting our criteria for a statistically significant difference (>2-fold change, <5% FDR) between the *cyr1Δ/Δ* mutant and its complemented derivative at pH 4 and pH 7, as well as statistically significant differences between pH 4 and pH 7 in the *cyr1* mutant and its complement. Download Table S2, XLSX file, 0.7 MB. Copyright © 2016 Hollomon et al. 2016 Hollomon et al. This content is distributed under the terms of the Creative Commons Attribution 4.0 International license.

10.1128/mSphere.00283-16.5 Table S3  Transcripts that differed in the *cyr1* mutant at both pH 4 and pH 7 in this study and in the study by Harcus and colleagues in 2004 (31). Download Table S3, XLSX file, 0.1 MB. Copyright © 2016 Hollomon et al. 2016 Hollomon et al. This content is distributed under the terms of the Creative Commons Attribution 4.0 International license.

10.1128/mSphere.00283-16.6 Table S4  Selected transcripts involved in central metabolism (values are expressed as the log_2_ fold difference for the *CYR1* mutant relative to the *cyr1Δ/Δ* mutant; positive values indicate expression was higher in the *CYR1* strain; ETC is an abbreviation for electron transport chain). Download Table S4, XLSX file, 0.03 MB. Copyright © 2016 Hollomon et al. 2016 Hollomon et al. This content is distributed under the terms of the Creative Commons Attribution 4.0 International license.

10.1128/mSphere.00283-16.7 Table S5  Gene ontology term enrichment analysis of genes differentially expressed in the presence and absence of the *CYR1* complemented strain at pH 7 and pH 4, as well as those differentially expressed between pH 4 and pH 7 in the *cyr1Δ/Δ* mutant and its complement. Download Table S5, XLSX file, 0.1 MB. Copyright © 2016 Hollomon et al. 2016 Hollomon et al. This content is distributed under the terms of the Creative Commons Attribution 4.0 International license.

**TABLE 1:** Selected transcripts dysregulated in the absence of Cyr1 during adaptation to neutral or low pH^a^

Gene	orf19designation	Log_2_ fold change for comparison of:		
		*CYR1* vs *cyr1*Δ/Δ strains at:		pH 7 vs pH 4 with*CYR1* strain
		pH 7	pH 4	
*HYR1*	orf19.4975	11.1	9.6	NS
*ECE1*	orf19.3374	9.9	6.7	NS
*UME6*	orf19.1822	9.2	6.9	NS
*RBT1*	orf19.1327	6.8	6.2	NS
*ALS3*	orf19.1816	6.0	4.8	NS
*SAP4*	orf19.5716	4.0	1.3	NS
*ALS4*	orf19.4555	−4.8	−3.0	NS
*SAP6*	orf19.5542	8.5	3.5	4.3
*SAP5*	orf19.5585	6.8	2.1	2.5
*IHD1*	orf19.5760	5.4	5.1	1.5
*HWP1*	orf19.1321	8.7	7.1	1.4
*HGC1*	orf19.6028	2.6	1.8	1.4
*NRG1*	orf19.7150	−2.3	−1.4	−1.1
*YWP1*	orf19.3618	−4.4	−4.0	−1.3

a Positive values for the *CYR1* versus *cyr1*Δ/Δ strain comparisons at pH 7 (neutral) and pH 4 (low) indicate that the indicated gene’s expression was higher in the *CYR1* strain. Positive values for the comparison of *CYR1* expression at pH 4 versus pH 7 indicate that expression was higher at pH 7. NS, no significant difference.

**TABLE 2:** Biological process gene ontology groups enriched in the *cyr1*Δ/Δ strain or the *CYR1*-complemented strain[Table-fn ngtab2.1]^*a*^

Process	Regulation in *cyr1*Δ/Δ mutant vs *CYR1*-complemented strain at:	
	pH 7	pH 4
Lipid catabolism	−	−
Carboxylic acid catabolism	−	−
Amino acid transport	−	−
Peptide transport	−	−
Tricarboxylic acid cycle	−	−
Electron transport chain	−	
Mitochondrion organization		−
Iron-sulfur cluster assembly	−	−
Oxygen transport	−	−
Pyruvate metabolism	+	+
Carbohydrate catabolic process	+	+
*N*-Acetylglucosamine catabolism	+	+
Pentose-phosphate shunt	+	+
Carbohydrate biosynthesis	+	+
Fluconazole transport	−	−
Biofilm formation	±	
Cell matrix adhesion	+	
Translation	+	+
Protein glycosylation	+	+
Sulfur compound metabolism	+	−
Protein folding	−	
Cellular heat acclimation	−	
Zinc ion homeostasis	−	
Copper ion transmembrane transport		−

a Processes that were upregulated (+) or downregulated (−) or contained a mix of upregulated and downregulated genes (±) in the *cyr1*Δ/Δ strain relative to the *CYR1*-complemented strain at the indicated pH.

Alongside morphology, many transcripts related to metabolism were differentially expressed between the *cyr1*Δ/Δ mutant and its complemented derivative at both pH 4 and 7 (Table 2; Tables S2 and S4 in the supplemental material). Consistent with observations made by Harcus and colleagues (31), transcripts encoding elements of the tricarboxylic acid (TCA) cycle were lower in the *cyr1*Δ/Δ mutant. In addition, we found significantly lower levels of transcripts corresponding to the glyoxylate shunt enzymes *ICL1* and *MLS1* and the pyruvate dehydrogenase complex (Table S4). Among the pathways that were significantly enriched among the transcripts that were lower in the *cyr1*Δ/Δ mutant were carboxylic acid catabolism, lipid catabolism, and amino acid and peptide transport (Table 2). For example, the mutant had lower levels of transcripts encoding *JEN1* and *JEN2*, which encode carboxylic acid transporters known to take up TCA cycle intermediates, and the latter of which was shown to be Cyr1 regulated by Harcus and colleagues (31, 32). GO terms associated with carbohydrate catabolism, *N*-acetylglucosamine catabolism, gluconeogenesis, and the pentose-phosphate shunt were significantly enriched among transcripts that were higher in the *cyr1*Δ/Δ null strain (Table 2; Table S4). In addition, transcripts related to hexose uptake were also higher, as were transcripts for alcohol dehydrogenases *ADH1* and *ADH2*, in the *cyr1* null strain. Although metabolic flux differences can only be inferred from transcriptional analyses, these data suggested to us that the *cyr1* null strain is metabolically more fermentative than its complement, whose transcriptional profile is more suggestive of oxidative phosphorylation being the dominant mode of central carbon metabolism.

GO term analysis of transcripts that were different between the *cyr1* mutant and its complemented derivative demonstrated enrichment in several other pathways (Table 2). The GO terms translation and protein glycosylation were enriched among the transcripts that were higher in the *cyr1* mutant. Cyclic AMP signaling has been previously observed to negatively regulate azole resistance, and fluconazole transport was enriched at both pH 4 and pH 7 among those transcripts that were lower in the *cyr1* null mutant (33). Transition metal homeostasis was observed in our analysis under both conditions; interestingly, zinc ion homeostasis met our cutoff at pH 4 but not pH 7, while copper ion transport was present in an inverse pattern in our GO analysis. As discussed below, some pathways were only modulated by Cyr1 at one pH level.

### Effects of pH on gene expression.

Fewer transcripts showed changed expression levels between pH 4 and pH 7 than between the mutant and the complemented strain. Changes in pH altered the gene expression of 640 transcripts in the mutant and 608 in the complement. However, apart from a core group of 214 genes, 820 genes showed significantly changed expression in one strain or the other (Fig. 5B). Of those 214 genes in the core group, however, 74 transcripts had inverse patterns of expression (expression went up in response to low pH in the mutant and down in response to low pH in the complemented strain, or vice versa). Of the genes that showed changed expression with pH changes in the complemented strain, 140 transcripts had similar patterns of expression between pH 4 and pH 7 in the *cyr1* null strain and 896 transcripts failed to change at all with pH or changed in the opposite direction in the *cyr1Δ/Δ* mutant strain*.*

We also performed a principal-component analysis (PCA) of the transcriptional response to changes in pH with and without intact cAMP signaling. PCA found little similarity in the pH responses between the *cyr1* null strain and its complemented derivative, and the effects we observed of pH on the transcriptome were similar in scope in the *cyr1*Δ/Δ mutant compared to the *CYR1* complemented strain (Fig. 5C). Given the very different responses to pH in these two strains, a transcriptional approach to determining the role of Cyr1 in the adaptation to a change in pH was complex and yielded results that were challenging to interpret. That being said, some, but not all, of the Cyr1-regulated morphology-associated transcript levels (*HWP1*, *YWP1*, *HGC1*, *SAP6*, *NRG1*, and *YWP1*) were lower at pH 4 than at pH 7, which is consistent with decreased Cyr1 activity at low pH (Table 1). These data are consistent with our model in which some of the differences between *C. albicans* at pH 4 and pH 7, particularly the morphological differences, are due, at least in part, to changes in Cyr1 activity.

We compared the transcripts that changed between pH 4 and pH 7 in the *CYR1*-complemented strain to those that were reported by Bruno et al. in 2010 (34), who described the transcriptional differences between cells grown at pH 4 versus pH 8 in M199 tissue culture medium. We identified 115 transcripts that were similarly changed at low versus high pH, with 66 exhibiting lower expression levels at low pH and 59 more highly expressed at low pH (see Table S6 in the supplemental material). This group of genes included genes for the pH-regulated cell wall glycosidases *PHR1* and *PHR2*, the transcription factor *RIM101*, as well as dicarboxylic acid transporters *JEN1* and *JEN2* and the multidrug resistance pump *MDR1*.

10.1128/mSphere.00283-16.8 Table S6  Genes that were significantly differentially expressed at different pHs in the *CYR1* complemented strain in this study and those identified by Bruno et al. in 2010 (34) to be differentially expressed in M199 at pH 4 versus pH 8. Download Table S6, XLSX file, 0.1 MB. Copyright © 2016 Hollomon et al. 2016 Hollomon et al. This content is distributed under the terms of the Creative Commons Attribution 4.0 International license.

Notably enriched GO terms of transcripts more abundant at pH 7 than at pH 4 in the *CYR1*-complemented strain included regulation of the response to stress, as well as cell adhesion, biofilm formation and adhesion, and the electron transport chain, particularly transcripts associated with complex I and amino acid transport (Table 3; Table S5). Enriched GO terms among the less abundant transcripts at pH 7 versus pH 4 included many involved in transport, such as those for transport of azole drugs (*MDR1* and *FLU1*), metals, hexoses, and organic anions. Among the transcripts that were lower at low pH, there was enrichment of those involved in sulfur amino acid and branched-chain amino acid biosynthesis (Table 4). Chitin catabolism genes were also enriched among pH 7-repressed transcripts, suggesting carbohydrate or cell wall requirements may be influenced by pH. Consistent with the model that pH reduces Cyr1 activity, some of the same GO term categories (amino acid transport, azole transport, and biofilm formation) were enriched among transcripts that were lower in cells grown at pH and in the *cyr1*Δ/Δ mutant cells.

**TABLE 3:** Biological process gene ontology groups enriched among the subset of transcripts that were higher at pH 7 than at pH 4 in the *CYR1*-complemented strain

Process	Genes in GO group
Regulation of response to stress	*SAP6*, *ALS1*, *SAP5*, *RIM101*, *PRA1*, *ACE2*, *HGT1*, *BCR1*, *CPH1*, *SRR1*, *AHR1*
Biofilm formation	*PHR1*, *ALS1*, *IPT1*, *TRY6*, *RIM101*, *TRY4*, *SUC1*, *ACE2*, *HWP1*, *HGC1*, *HSP104*, *WOR1*, *QDR1*, *BCR1*, *CPH1*, *AHR1*, *ADH5*
Cell adhesion	*PHR1*, *ALS1*, *TRY6*, *RIM101*, *PRA1*, *AAH1*, *TRY4*, *SUC1*, *ACE2*, *HWP1*, *WOR1*, *BCR1*, *AHR1*
Electron transport chain	*NAD5*, *NAD3*, *NAD4*, *COX2*, *NAD2*, *NAD6*
Amino acid transport	*AGP2*, *GAP6*, *CAN2*, *GAP2*, *PUT4*, *DIP5*, *GNP3*, *GAP4*, *HNM4*

**TABLE 4:** Biological process gene ontology groups enriched among the subset of transcripts that were lower at pH 7 than at pH 4 in the *CYR1*-complemented strain

Process	Genes in GO group
Azole transport	*FLU1*, *MDR1*
Metal ion transport	*ZRT2*, *TRK1*, *FTR1*, *FET3*, *CTR1*, *MAC1*, *PHO87*, *FET99*, *HAK1*, *FRE10*
Hexose transport	*HGT8*, *HGT10*, *NAG3*, *NAG4*, *HGT19*, *HGT7*, *HGT17*
Organic anion transport	*JEN2*, *TPO5*, *GIT4*, *GNP1*, *ALP1*, *JEN1*
Chitin catabolic process	*CHT2*, *CHT1*
Branched-chain amino acid biosynthesis	*ILV5*, *BAT21*, *BAT22*, *PDC11*
Sulfur amino acid biosynthesis	*MET16*, *ECM17*, *MET2*, *MET1*, *MET15*, *MET3*

Some pathways changed significantly in opposing directions with pH changes when the *cyr1* null and complemented strains were compared. Notable among these were genes involved in sulfate assimilation (*MET3*, *MET14*, *MET16*, and *MET10*, which encode enzymes involved in the conversion of sulfate to hydrogen sulfide), which increased at pH 4 in the mutant and decreased in its complement (Table 2). A number of transcripts encoded on the mitochondrial genome also showed Cyr1-dependent changes in expression with pH changes; transcripts encoding components of the electron transport chain significantly decreased in abundance in the complemented strain at low pH, but in the mutant many of these components showed inverse patterns of expression (see Table S4).

We examined the data for transcripts of genes known to be involved in the pH response. *PHR1* and *PHR2* are two pH-responsive transcripts that encoded cell wall glycosidases that are reciprocally expressed at low and neutral pH, with *PHR1* showing higher expression at pH 7, and they have previously been observed to be dysregulated in the absence of Cyr1 (31). *ATO1* is an ammonium exporter that participates in medium alkalinization and autoinduction of hyphal growth (35, 36). Of the transcripts that changed significantly in similar directions with pH in both the mutant and complemented strain were *PHR1* and *PHR2*, as well as *ATO1*; however, the magnitude of these changes was lower in the absence of Cyr1. *ATO1* was increased 71-fold in the complemented strain at pH 7 versus that at pH 4 but only 18-fold in the mutant, and *PHR1* was similarly induced 11-fold in the mutant and 21-fold in the complemented strain under neutral pH conditions compared to acidic pH. *PHR2* decreased 2.9- and 6.8-fold in the mutant and complemented strain, respectively, at pH 7 versus pH 4.

*RIM101* was more highly expressed at pH 7 than pH 4 in the complemented strain, but it was not significantly changed in the *cyr1* null strain with pH changes. A number of genes whose expression changed with pH have previously been described to be regulated by Rim101. In total, 41 transcripts annotated as Rim101 regulated underwent statistically significant changes in either the presence or absence of Cyr1 (Table 5). Thirty-one transcripts annotated as Rim101 regulated by CGD were altered in expression in the *CYR1* complemented strain (including *RIM101*), and 25 were altered in the mutant strain (37). Of these, however, only 16 changed in both strains.

**TABLE 5:** Transcripts significantly different based on RNA-seq data that are annotated as Rim101-regulated in the *Candida* Genome Database[Table-fn ngtab5.1]^*a*^

Presence of *CYR1* and gene name	orf19designation	Log_2_ fold change in expressionat pH 7 vs pH 4 in:	
		*CYR1*-complementedstrain	*cyr1*Δ/Δ strain
Expressed only when *CYR1* is absent			
* MUP1*	orf19.5280	NS	1.9
* PHO113*	orf19.2619	NS	−1.8
* RBR3*	orf19.5124	NS	−1.2
* RBT1*	orf19.1327	NS	−1.1
* GIT1*	orf19.34	NS	−3.7
* PGA10*	orf19.5674	NS	−2.2
* PGA23*	orf19.3740	NS	−1.4
* PHO112*	orf19.3727	NS	−1.8
Expressed only when *CYR1* is present			
* RIM101*	orf19.7247	2.1	NS
* HOL4*	orf19.4546	1.8	NS
* CDR11*	orf19.918	1.8	NS
* FRP2*	orf19.7112	1.5	NS
	orf19.7566	1.3	NS
* DIP5*	orf19.2942	1.3	NS
* ECM21*	orf19.4887	1.3	NS
* PHO8*	orf19.984	1	NS
* PGA7*	orf19.5635	−1.1	NS
* KRE6*	orf19.7363	−1.1	NS
* CRH12*	orf19.3966	−1.2	NS
* PTR22*	orf19.6937	−1.7	NS
* GNP1*	orf19.1193	−2.1	NS
* FRE10*	orf19.1415	−3	NS
* RBE1*	orf19.7218	−3.2	NS
Expressed in both strains[Table-fn ngtab5.2]^*b*^			
* CFL2*	orf19.1264	2.3	3.8
* PUT1*	orf19.4274	1	−1.1
	orf19.851	−1.1	−3.3
* CTR1*	orf19.3646	−1.3	−1.6
* RBR2*	orf19.532	−1.5	−1.5
	orf19.7077	−1.7	−2.4
* OPT3*	orf19.3749	−2.3	−2.7
* FET99*	orf19.4212	−2.7	−9.5
* JEN1*	orf19.7447	−2.9	−1.3
* CRZ2*	orf19.2356	−3.7	−6
* RBR1*	orf19.535	−6.8	−7.1

a Positive values indicate expression was higher at pH 7. NS, no significant difference.

b The following *Rim101*-regulated transcripts were differentially expressed in response to pH 7 versus pH 4, but expression was not different between the *CYR1*-complemented and *cyr1*Δ/Δ strains at pH 7: *FRP1*, *HMX1*, *SKN1*, *ENA2*, *PHO89*, *PHO87*, and *PHR2*.

Using NanoString nCounter technology, we used a code set that included morphology genes and metabolism and used it to interrogate RNA from cells grown in a separate experiment on a separate day than the RNA-Seq analysis. The impact of Cyr1 was strongest in the regulation of the morphological genes, but there was a pH effect on hypha- and yeast-associated transcripts in the complemented strain, with the exception of *HYR1*, which increased in abundance at low pH. In the strain with intact Cyr1 signaling, we observed the induction of *PHR1* and *PHR2* at neutral and low pH, respectively, and the expression pattern of these transcripts was notably muted in the absence of Cyr1 (Table 6; see also Table S7 in the supplemental material).

10.1128/mSphere.00283-16.9 Table S7  Transcription of the *cyr1*Δ/Δ mutant and its complement, performed by analysis of RNA using the Nanostring nCounter technology. Download Table S7, XLSX file, 0.1 MB. Copyright © 2016 Hollomon et al. 2016 Hollomon et al. This content is distributed under the terms of the Creative Commons Attribution 4.0 International license.

**TABLE 6:** Selected morphology- and pH-related transcription changes

Gene	Log_2_ fold change in transcription for:		
	*CYR1* vs *cyr1*Δ/Δ strain at:		pH 7 vs pH 4 in*CYR1* strain
	pH 7	pH 4	
*HWP1*	9.0	9.6	0.4
*HYR1*	9.0	10.6	−1.5
*ECE1*	8.3	8.6	1.0
*SAP4*	3.8	2.4	1.2
*YWP1*	−4.3	−2.6	−1.3
*ALS4*	−6.3	−2.5	−0.8
*NRG1*	−1.8	−0.7	−1.2
*PHR1*	2.6	3.0	3.5
*PHR2*	−0.2	1.4	−4.5

## DISCUSSION

In this work, we set out to determine if changes in cAMP signaling participate in the repression of filamentous growth induced by a low-pH environment in *C. albicans*. We found that hyphal growth was antagonized by low pH under medium conditions that stimulated hyphal growth in a Ras1- and Cyr1-dependent manner. Low extracellular pH resulted in Cyr1-dependent increases in Ras1 proteolysis and GTP binding, which were both rapid and sustained, but neither increased GTP binding nor cleavage was responsible for the effect of low pH on morphology. Filamentous growth could be rescued in a *cyr1* null mutant by dbcAMP at both low and neutral pHs, which supports a model in which low pH acts at least partially upstream of Cyr1. Under our medium conditions, intracellular pH was reduced by extracellular pH, and we hypothesize that this reduces the availability of bicarbonate, a Cyr1-stimulatory factor. Analysis of the transcriptome in the presence and absence of intact cAMP signaling at acid and neutral pH painted a complicated picture, with Cyr1-dependent and -independent changes in response to pH and differences in pH response that were dependent on whether cAMP signaling was intact. Consistent with Cyr1 playing a role in the adaptation to low and neutral pH, Cyr-regulated transcripts related to morphology were altered in expression by changes in pH, and the expression levels of the pH-specific genes *PHR1* and *PHR2* were substantially greater in the presence of Cyr1.

Cytosolic pH is the cumulative result of buffering by a large number of different molecules (charged amino acid side chains, free amino acids, glycolytic intermediates, and others), and the activity of plasma membrane and vacuolar ATPases (V-ATPases) and proton pumps (38). In *Saccharomyces*, V-ATPases are regulated by Ras and phosphoinositides, and V-ATPases themselves reciprocally regulate Ras (39–41). Intracellular pH in *Saccharomyces cerevisiae* has been shown to be reduced in the presence of citric acid/phosphate buffer at low pH, and in *C. albicans*, growth in medium favoring yeast growth due to low-pH medium has been described to result in cytosolic acidification compared to an otherwise identical neutral hypha-inducing medium, and the dynamics of changes in intracellular pH are different between cells growing as hyphae and those growing as yeast (42, 43). Despite mechanisms to modulate intracellular pH, our findings using pHluorin-expressing cells showed that extracellular pH has rapid and prolonged effects on intracellular pH. These findings mirror observations by Kaur and colleagues (43), who saw a lower pH_i_ in cells in pH 4.5 medium than in cells at pH 6.5, using [^14^C]propionate distribution as an indicator of pH_i_. In the *tetO-NRG1* strain, the external pH still impacted cytosolic pH to a similar extent, suggesting to us that extracellular pH, as well as the hyphal growth transcriptional network itself, make independent but additive contributions to intracellular pH.

In *C. albicans*, bicarbonate is a Ras1-independent activator of Cyr1, and its availability is determined by the carbonic anhydrase Nce103, its substrate CO_2_, and pH (29). As bicarbonate exists in equilibrium with its conjugate acid, carbonic acid, a reduction in pH would drive that equilibrium toward the conjugate acid, depleting a Cyr1 activator. CO_2_ has been demonstrated to control hyphal morphology through Cyr1, and mutation of the specific residues of Cyr1 responsible for bicarbonate sensing resulted in a diminished ability to form filaments under high CO_2_ (which is Ras independent) but a normal ability to do so in response to serum (which is Ras dependent) (44). White-opaque switching has also been shown to be regulated by CO_2_ in a Cyr1-dependent, but also partially Ras1-dependent, fashion (45). Taken together, these observations suggest that Cyr1 integrates Ras1-dependent and -independent inputs, with different phenotypes requiring different degrees of contribution from each source.

pH has been postulated to regulate bicarbonate-sensitive adenylate cyclases in a number of systems (development of mammalian spermatozoa during capacitation being the classic example), but to our knowledge, the extent of perturbation of pH_i_ necessary to alter adenylate cyclase output has not been empirically determined *in vivo* (46–48). We observed a shift in strain SC5314 from pH_i_ 8.0 to pH_i_ 6.6 between neutral and acidic culture conditions, and it is plausible that this represents a significant change in the bicarbonate/carbonic acid equilibrium and Cyr1 activation. This equilibrium has a theoretical pK_a_ of 3.6 in the absence of carbon dioxide, but likely a much higher effective pK_a_ *in vivo*, as CO_2_ is generated through oxidative metabolism. Using the carbonic acid/bicarbonate pK_a_ for mammalian blood chemistry (6.4), one can calculate the fraction of bicarbonate predicted to exist in the unprotonated form at our measured pH_i_ levels. In cells grown in YNBNC at pH 7, where the pH_i_ was 8.0, 98% of the bicarbonate would be in its unprotonated from, whereas in cells grown at pH 4, the pH_i_ was 6.9, where 80% of the bicarbonate would be in its unprotonated form. Alternatively, it is possible that the change in pH alone is necessary to alter cyclase activity, as reports have suggested that soluble adenylate cyclases favor neutral to basic pHs for catalysis, although these experiments were conducted with crude cellular extracts that likely contained bicarbonate, and it may not be possible to extricate the role of pH *per se* from the availability of bicarbonate (49, 50).

From the massive transcriptional changes induced by pH for genes associated with diverse cellular processes such as hyphal growth, cell wall architecture, and metabolism, it is clear that ambient pH is an important regulator of *C. albicans* physiology (34, 51). *C. albicans* has been shown to actively modulate the pH of its environment; work from the Lorenz group has illuminated a mechanism by which *C. albicans* induces filamentation by manipulating the environmental pH through secretion of basic products of metabolism (35). *C. albicans* Rim101 was initially characterized as an activator of hyphal growth in response to high pH as a stimulus (7, 25, 52, 53). Low pH can be thought of as the absence of high pH, or a stimulus unto itself, and analysis of our data alongside published work is consistent with a model that integrates both of these elements. Consistent with the model that there exist multiple pH-sensitive regulators, in our dbcAMP rescue assays more cells grew as hyphae at pH 7 than pH 4 in the presence of dbcAMP, suggesting that Cyr1 is only one layer of morphological regulation by pH.

Growth at low pH under our conditions resulted in a population that was predominantly pseudohyphal, which is thought to represent an intermediate state between yeast and hyphal growth (54). Thus, we argue that our data suggest that low pH results in an intermediate level of cAMP output, where the hypha-activating cues are able to turn on the pathway sufficiently to support pseudohyphal growth, but low pH prevents it from being turned on to a sufficient extent to permit true hyphal growth. This intermediate state is phenotypically very different from the total absence of cAMP signaling that we saw in the *cyr1* null mutant, and it had a remarkably different transcriptional profile; in this state, we are able to see the effects of other regulators, such as Rim101, in the transcriptional data that are washed out in the complete absence of Cyr1. A previous transcriptional analysis of the role of Rim101 in the adaptation to acidic and alkaline pH found that Rim101 regulated some, but not all, genes that changed with pH and that Rim101 regulated a number of genes independently of pH (51). Via synthesis of this finding with the fact that the effect of pH was difficult to discern through the overwhelming effect of Cyr1 in our data, we are able to infer that the genetic basis of the response to pH is complicated, and we suggest deletion of single regulators cannot fully explain changes in the transcriptome induced by changes in pH.

With respect to the role of pH in the course of human disease, acidic pH is an environmental condition confronted by *C. albicans* in the phagolysosome, where alkalinization by *C. albicans* metabolism is thought to facilitate survival of the fungus through the autoinduction of hyphal growth (35, 36, 55). There is precedent for inappropriate activation of cAMP signaling preventing the adaptation to a low-pH environment; Wilson and colleagues (56) previously described cAMP hyperactivation, due to the loss of the major cAMP phosphodiesterase, rendered *C. albicans* sensitive to acidic pH, with a *pde2* null mutant unable to grow at pH 2.5. This suggests that downregulation of cAMP signaling participates in the adaptation to low-pH environments. Cyr1 has been demonstrated to be necessary for pathogenesis in immunocompetent mice in a model of disseminated candidiasis, and under our experimental conditions, the *ATO1*-encoded ammonium exporter through which *C. albicans* alkalinizes its environment, in addition to being regulated by pH itself was altered in its regulation in the absence of Cyr1 (35, 36). Notably, two transcripts that we saw prominently induced by low pH, *JEN1* and *JEN2*, have been shown to be induced upon phagocytosis by neutrophils (32). Based on these observations, the regulation of Cyr1 by pH may play a role in the adaptation to the phagocytic vacuole. Additionally, *Candida* adapts to extremely low pH values in the stomach in the course of intestinal commensalism, which can be the source from which *C. albicans* disseminates in invasive disease.

Vulvovaginal candidiasis (VVC) is one of the most common fungal infections in humans, affecting most women at some point over the course of their lives, and represents a context in which acidic pH is a prominent feature of the environment. Vaginal pH in the context of health is low (<4.5); however, unlike other forms of vaginitis, VVC is not associated with alkalization of the vagina, and diagnostically, an acidic vaginal pH is consistent with VVC (2). Histological evidence indicates filamentous forms are present in VVC, and a yeast-locked strain is defective in a rat model of VVC (57). Notably, a *cyr1* null strain is also defective in the ability to persist in the murine vaginal mucosa compared to its complemented derivative, signifying the importance of this signaling component in this niche (13). Growth of wild-type *Candida* in association with vaginal epithelial cells produced transcriptional changes in many of the same pathways we observed in our transcriptional analysis (glucose and GlcNAc metabolism, Efg1), suggesting that cAMP plays a role in this environment (58). Taken together, these observations strongly imply that there are other factors apart from pH that govern hyphal growth in the host, including the existence of other stimuli that supersede the pH signal or alternate pathways capable of inducing hyphal growth in a cAMP- and Rim101-independent fashion. We suggest that the integration of pH into the complex decision-making circuits governing *Candida* morphology merits further study.

## MATERIALS AND METHODS

### Strains and growth conditions.

All *C. albicans* strains were streaked from frozen stocks maintained at −80°C onto YPD (1% yeast extract, 2% peptone, 2% glucose) plates, incubated at 30°C for 24 to 48 h, then stored at room temperature. All strains used in this study can be found in Table S1 in the supplemental material.

### Strain construction.

The BWP17 *rim101Δ/Δ* strain and BWP17 *rim101+/Δ* strain were constructed as previously described, using deletion amplicons amplified from pGEM-HIS1 and pRS-ARG4 with flanking homology to the *RIM101* open reading frame (52, 59). Cells were transformed via electroporation, selected on YNB medium lacking the appropriate amino acid(s), and confirmed using PCR with primers flanking the *RIM101* locus. The SC5314 *rim101Δ/Δ* mutant was generated with the transient CRISPR-Cas9 system (60). Briefly, strain SC5314 was cotransformed with the *RIM101*-NAT deletion construct (3 μg), the *CaCAS9* cassette (1 μg), and the sgRNA cassette (1 μg) by using the lithium acetate transformation method (61). We used the following sgRNA *RIM101* guide RNA sequence, published by Vyas et al. (2015) to generate the deletion mutant: AGCAAAAGCTGCTGGCTTGG (62).

### Morphological assessment.

For morphological assessment, overnight cultures were grown in YPD and used to inoculate YNBNP medium (as described by Piispanen et al. [22]), or YNBNC (0.67% yeast nitrogen base–5 mM *N*-acetylglucosamine–0.2% glucose–50 mM citrate at pH 4 or pH 7) to a final density of 10^6^ cells per ml. YNBNC was prepared by addition of YNB salts without amino acids (RPI Corp.), GlcNAc (Alfa Aesar) from a 1 M stock solution prepared in water, and glucose from a 20% (wt/vol) solution in water and adjustment to final volume with distilled water. This medium was adjusted to pH 7 with strong base (NaOH) to make YNBN (pH 7-adjusted) medium and filter sterilized. YNBNC was made by addition of 50 mM citrate at the indicated pH from a sterile 1 M citrate buffer stock to YNBN medium. Five-milliliter cultures were subsequently incubated at 37°C in tubes on a roller drum for 3 h, an aliquot of culture was transferred to a slide, and morphology was assessed via differential interference contrast (DIC) microscopy.

### Growth rate experiments.

Growth was assayed by dilution of overnight cultures to an optical density at 600 nm (OD_600_) of 0.05, and optical density was measured over time using a Spectronic 20 D spectrophotometer.

### dbcAMP experiments.

Cells were inoculated into the indicated medium at a density of 5 × 10^5^ cells per ml from a YPD overnight culture. Medium amended with 10 mM dbcAMP from a 100 mM stock solution of dbcAMP (D0627; Sigma) in water. Cultures were incubated at 37°C for 3 h in glass-bottom 12-well dishes and then fixed with 0.37% formaldehyde, and morphological assessments by DIC microscopy were performed on an inverted microscope.

### Western blot analysis of Ras1.

Overnight cultures in YPD were washed once in target medium and inoculated into YNBNC at 5 × 10^7^ cells per ml, and cultures were incubated as described above. After 3 h, cells were pelleted by centrifugation at 4,500 × *g* for 5 min and snap-frozen in liquid nitrogen. Pellets were thawed on ice, washed once in homogenization buffer (10 mM Tris [pH 7.4], 150 mM NaCl, 5 mM EDTA, 2× Halt protease inhibitor cocktail [Fisher], and 10% [wt/vol] sucrose), resuspended in homogenization buffer, and lysed via bead beating. Protein concentrations of whole-cell lysates were assessed with the Bradford assay (Bio-Rad Quick Start with Bradford dye reagent). SDS-PAGE and Western blotting were conducted as previously described, using anti-RAS clone 10 mouse monoclonal antibody (EMD Millipore).

### Analysis of Ras1-GTP binding state.

The Pierce Active Ras pulldown kit was used for analysis of Ras1-GTP binding, and whole-cell lysates were prepared as for the Western blotting assays, with the replacement of homogenization buffer (HB) or with lysis-binding-wash (LBW) buffer (25 mM Tris-HCl [pH 7.2], 150 mM NaCl, 5 mM MgCl_2_, 1% NP-40, and 5% glycerol) supplemented with 2× Halt protease inhibitor cocktail. Two hundred micrograms of total protein was incubated with RBD-conjugated agarose and eluted by boiling in Laemmli sample buffer. The eluate as well as the input lysate were subjected to Western blotting as described above.

### pHluorin analysis of intracellular pH.

Cells expressing the *Candida*-optimized pHluorin allele were analyzed as described in reference 30. Briefly, cells were grown as described above (in YNBN or YNBNC at pH 37°C) and with YNB LoFlo (catalog number CYN6201; Formedium), a low-fluorescence variant of YNB salts used to replace YNB salts), and the cytosolic pH was determined by measuring florescence at an emission wavelength at 518 nm and excitation wavelengths of 405 nm and 485 nm. Permeabilized control cells were transferred to calibration buffers to generate a standard curve, and the intracellular pH for experimental cells was extrapolated from that curve.

### nCounter transcriptional analysis.

YNBNC was inoculated with 5 × 10^7^ cells per ml as described above, and cultures were grown for 4 h at 37°C. Samples were prepared in duplicate, and cells were pelleted by centrifugation at 4,500 × *g* for 5 min and snap-frozen in liquid nitrogen. RNA was extracted with the Epicentre MasterPure yeast RNA purification kit (MPY03010), and 70 ng total RNA was hybridized to the NanoString probe set and quantified using the nCounter platform. Counts were normalized to total signal for each given sample, and replicates were averaged.

### RNA-Seq.

For RNA-Seq, YNBNC medium was inoculated with 5 × 10^7^ cells per ml as described above, and cultures were grown for 4 h at 37°C. Cells were pelleted by centrifugation at 4,500 × *g* for 5 min and snap-frozen in liquid nitrogen. RNA was extracted with the Epicentre MasterPure yeast RNA purification kit (MPY03010). RNA quality and quantity were assessed by using a fragment analyzer (Advanced Analytical, Ankeny, IA) and Qubit (Invitrogen, Carlsbad, CA), respectively. mRNA was enriched by hybridization to oligo(dT) beads. Directional RNA-Seq libraries were prepared with TruSeq stranded mRNA library prep chemistry with unique TruSeq indices, using an automated liquid-handling system. Libraries were pooled and sequenced on a NextSeq500 instrument, using 2× 75-bp paired-end sequencing (a high-output flow cell). Raw reads were processed using the CLC Genomics Workbench platform (v. 8.5.1) and the default parameter settings installed by the manufacturer. All sequences were trimmed and mapped to the SC5314 reference genome (version A21-s02-m09-r04; http://www.candidagenome.org) and with the use of the RNA-Seq analysis tool, and mapped reads were normalized to control for any differences in library size by using the commands “calcNormFactors,” “estimateCommonDisp,” and “estimateTagwiseDisp” with default settings in the edgeR package (v. 3.14.0). 

The full Gene Ontology annotation was used for GO term analysis. The gene association file, created 19 September 2016, was downloaded from the CGD website (http://www.candidagenome.org), and only annotations assigned to *C. albicans* (taxon 5476) were used. In total, 6,313 unique genes had at least one associated GO term and served as the background distribution of observed gene ontologies for the *C. albicans* genome in this study. GO enrichment analysis of the comparisons between the WT and the *cyr1* mutant at pH 4 and pH 7 was evaluated using an R script (GOstats.R, within bioconductor), in which the GSEAGOHyperGParams function was used for calculating a Bonferroni-corrected *P* value with a cutoff of 0.05 to determine significant GO term enrichment in the categories Biological Process, Cellular Component, and Molecular Function. The 100 most significantly enriched terms were retained for analysis, followed by removal of similar terms.

### Accession number(s).

The raw and processed RNA-Seq data have been deposited into NCBI Gene Expression Omnibus under GenBank accession number GSE86540.
