# Supplementary figures and images for: Global Role of Cyclic AMP Signaling in pH-Dependent Responses in Candida albicans
Source: mSphere. 2016 Nov 30;1(6):e00283-16. doi: 10.1128/mSphere.00283-16 (PMC5137381; doi:10.1128/mSphere.00283-16)

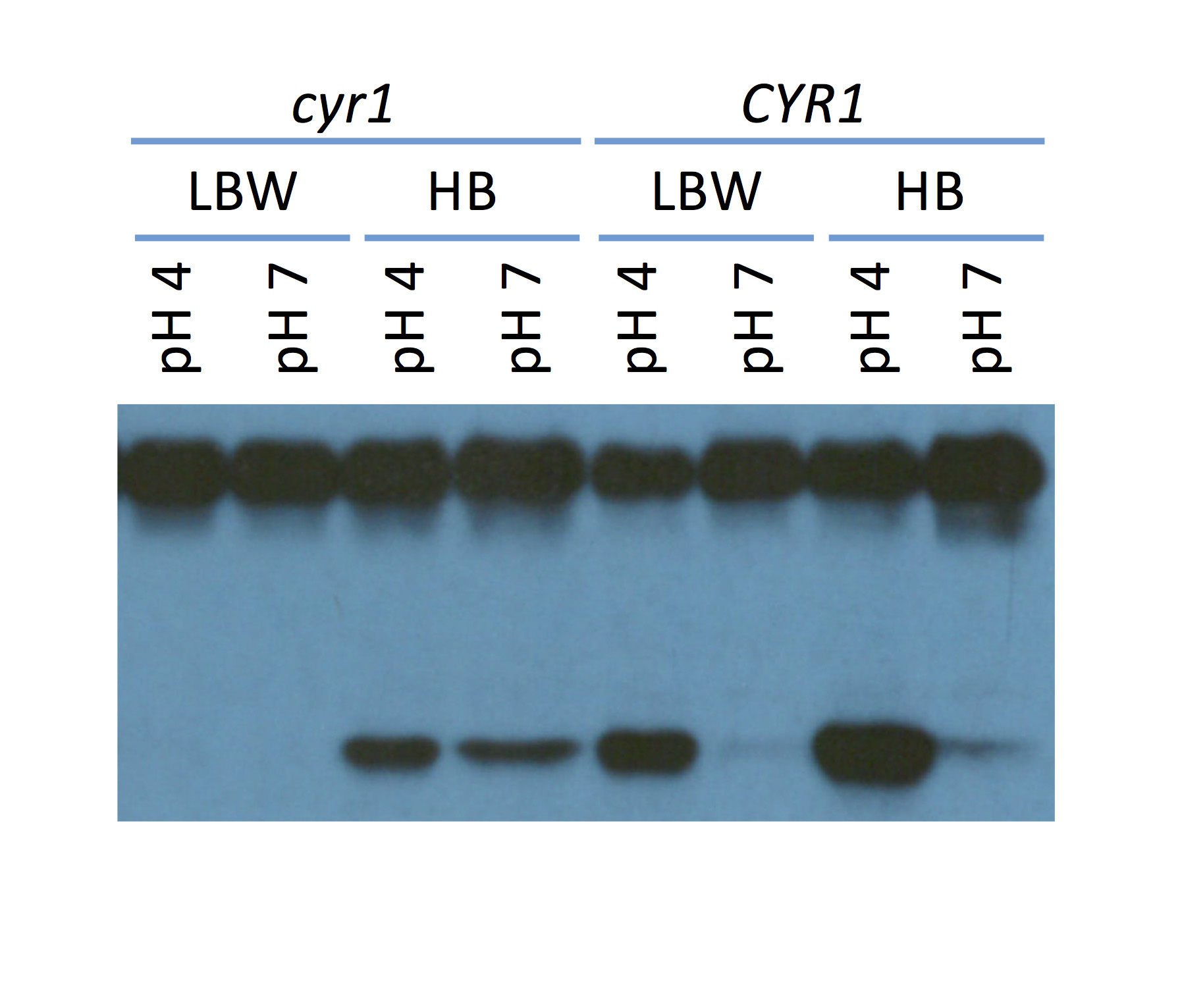

Supplement: Figure S1 [file sph006162187sf1.jpg]

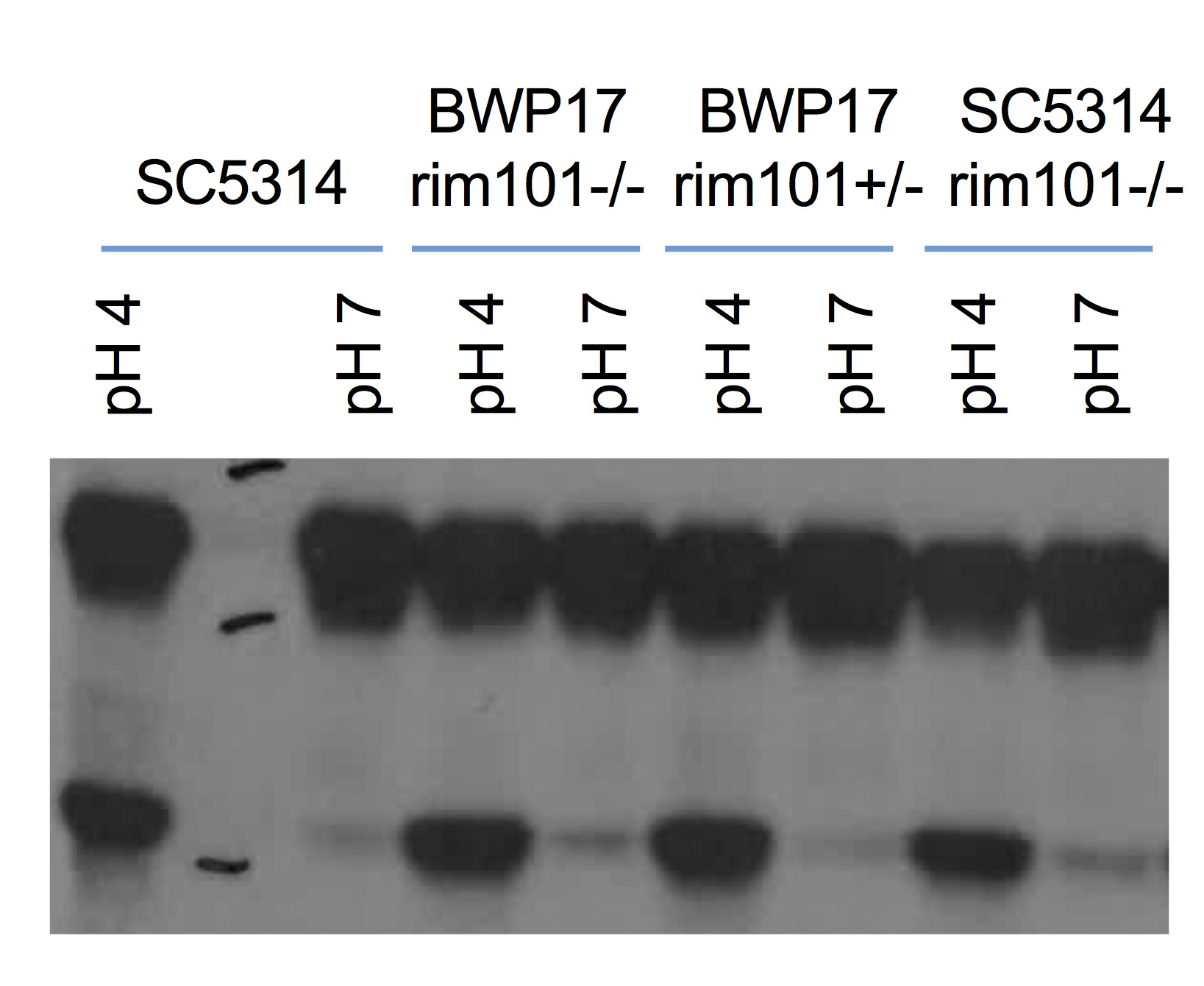

Supplement: Figure S2 [file sph006162187sf2.jpg]
