# Supplementary material for: Global Role of Cyclic AMP Signaling in pH-Dependent Responses in Candida albicans
Source: mSphere. 2016 Nov 30;1(6):e00283-16. doi: 10.1128/mSphere.00283-16 (PMC5137381; doi:10.1128/mSphere.00283-16)
Supplement: Table S1 [file sph006162187st3.docx]

| Strain Name | Genotype | Lab stock # | Reference |
| --- | --- | --- | --- |
| SC5314 | Prototrophic clinical isolate | DH35 | (1) |
| *ras1∆/∆* | *ura3::λimm434/ura3::λimm434 ras1::hisG/ras1::hisG:URA3* | DH483 | (2) |
| *RAS1* | *ura3::λimm434/ura3::λimm434 ras1::hisG/ras1::hisG:RAS1-URA3* | DH1385 | (3) |
| *ras1Δ200-220* | *ura3::λimm434/ ura3::λimm434 ras1::hisG /ras1::hisG- ras1Δ200-220* | DH1878 | (4) |
| *ras1G13V* | *ura3::λimm434/ ura3::λimm434 ras1::hisG /ras1::hisG::ras1-G13V-URA3* | DH1658 | (3) |
| *cyr1∆/∆* | *ura3::λimm434/ura3::λimm434::URA3 arg4::hisG/arg4::hisG his1::hisG/his1::hisG cyr1::HIS1/cyr1::ARG4* | DH2185 | (5) |
| *CYR1* | *ura3::λimm434/ura3::λimm434::URA3-CYR1 arg4::hisG/arg4::hisG his1::hisG/his1::hisG cyr1::HIS1/cyr1::ARG4* | DH2186 | (5) |
| *ira2∆/∆* | *ura3::λimm434/ura3::λimm434::URA3 arg4::hisG/arg4::hisG his1::hisG/his1::hisG ira2::HIS1/ira2::ARG4* | DH2240 | (5) |
| *tetO-UME6* | *ade2::hisG/ade2::hisG ura3::λimm434/ura3::λimm434::URA3-tet-O-UME6 ENO1/eno1::ENO1-tetR-ScHAP4AD-3XHA-ADE2* | DH2317 | (6) |
| *tetO-NRG1* | *ade2::hisG/ade2::hisG ura3::λimm434/ura3::λimm434::URA3-tet-O-NRG1 ENO1/eno1::ENO1-tetR-ScHAP4AD-3XHA-ADE2* | DH2315 | (7) |
| BWP17 *rim101∆*/*RIM101* | *ura3::λimm434/ura3::λimm434 arg4::hisG/arg4::hisG his1::hisG/his1::hisG rim101::HIS1/RIM101* | DH2673 | This study. |
| BWP17 *rim101∆*/*∆* | *ura3::λimm434/ura3::λimm434 arg4::hisG/arg4::hisG his1::hisG/his1::hisG rim101::HIS1/rim101::ARG4* | DH2674 | This study. |
| SC5314 *rim101∆*/*∆* | *rim101::NAT/rim101::NAT* | DH2670 | This study. |

1. Gillum AM, Tsay EY, Kirsch DR. Isolation of the *Candida albicans* gene for orotidine-5'-phosphate decarboxylase by complementation of *S. cerevisiae* ura3 and E. coli pyrF mutations. Mol Gen Genet. 1984;198(2):179-82. PubMed PMID: 6394964.

2. Leberer E, Harcus D, Dignard D, Johnson L, Ushinsky S, Thomas DY, Schroppel K. Ras links cellular morphogenesis to virulence by regulation of the MAP kinase and cAMP signalling pathways in the pathogenic fungus *Candida albicans*. Mol Microbiol. 2001;42(3):673-87. PubMed PMID: 11722734.

3. Piispanen AE, Bonnefoi O, Carden S, Deveau A, Bassilana M, Hogan DA. Roles of Ras1 membrane localization during *Candida albicans* hyphal growth and farnesol response. Eukaryot Cell. 2011;10(11):1473-84. Epub 2011/09/13. doi: 10.1128/EC.05153-11. PubMed PMID: 21908593; PMCID: 3209056.

4. Piispanen AE, Grahl N, Hollomon JM, Hogan DA. Regulated proteolysis of *Candida albicans* Ras1 is involved in morphogenesis and quorum sensing regulation. Molecular Microbiology. 2013;89(1):166-78. doi: 10.1111/mmi.12268.

5. Grahl N, Demers EG, Lindsay AK, Harty CE, Willger SD, Piispanen AE, Hogan DA. Mitochondrial Activity and Cyr1 Are Key Regulators of Ras1 Activation of *C. albicans* Virulence Pathways. PLoS Pathog. 2015;11(8):e1005133. doi: 10.1371/journal.ppat.1005133. PubMed PMID: 26317337; PMCID: PMC4552728.

6. Johnston DA, Tapia AL, Eberle KE, Palmer GE. Three prevacuolar compartment Rab GTPases impact *Candida albicans* hyphal growth. Eukaryotic cell. 2013;12(7):1039-50. doi: 10.1128/EC.00359-12. PubMed PMID: 23709183; PMCID: PMC3697461.

7. Peters BM, Palmer GE, Nash AK, Lilly EA, Fidel PL, Jr., Noverr MC. Fungal morphogenetic pathways are required for the hallmark inflammatory response during *Candida albicans* vaginitis. Infect Immun. 2014;82(2):532-43. doi: 10.1128/IAI.01417-13. PubMed PMID: 24478069; PMCID: PMC3911367.
